# Neutrophil Migratory Patterns: Implications for Cardiovascular Disease

**DOI:** 10.3389/fcell.2022.795784

**Published:** 2022-03-02

**Authors:** Albert Dahdah, Jillian Johnson, Sreejit Gopalkrishna, Robert M. Jaggers, Darren Webb, Andrew J. Murphy, Nordin M. J. Hanssen, Beatriz Y. Hanaoka, Prabhakara R. Nagareddy

**Affiliations:** ^1^ Department of Surgery, Division of Cardiac Surgery, The Ohio State University Wexner Medical Center, Columbus, OH, United States; ^2^ Division of Immunometabolism, Baker Heart and Diabetes Institute, Melbourne, VIC, Australia; ^3^ Amsterdam Diabetes Centrum, Internal and Vascular Medicine, Amsterdam UMC, Amsterdam, Netherlands; ^4^ Department of Internal Medicine, Division of Rheumatology, The Ohio State University Wexner Medical Center, Columbus, OH, United States

**Keywords:** neutrophils, margination, demargination, migration, cardiovasclar disease, catecholamine stress

## Abstract

The body’s inflammatory response involves a series of processes that are necessary for the immune system to mitigate threats from invading pathogens. Leukocyte migration is a crucial process in both homeostatic and inflammatory states. The mechanisms involved in immune cell recruitment to the site of inflammation are numerous and require several cascades and cues of activation. Immune cells have multiple origins and can be recruited from primary and secondary lymphoid, as well as reservoir organs within the body to generate an immune response to certain stimuli. However, no matter the origin, an important aspect of any inflammatory response is the web of networks that facilitates immune cell trafficking. The vasculature is an important organ for this trafficking, especially during an inflammatory response, mainly because it allows cells to migrate towards the source of insult/injury and serves as a reservoir for leukocytes and granulocytes under steady state conditions. One of the most active and vital leukocytes in the immune system’s arsenal are neutrophils. Neutrophils exist under two forms in the vasculature: a marginated pool that is attached to the vessel walls, and a demarginated pool that freely circulates within the blood stream. In this review, we seek to present the current consensus on the mechanisms involved in leukocyte margination and demargination, with a focus on the role of neutrophil migration patterns during physio-pathological conditions, in particular diabetes and cardiovascular disease.

## Introduction

**FIGURE 1:**
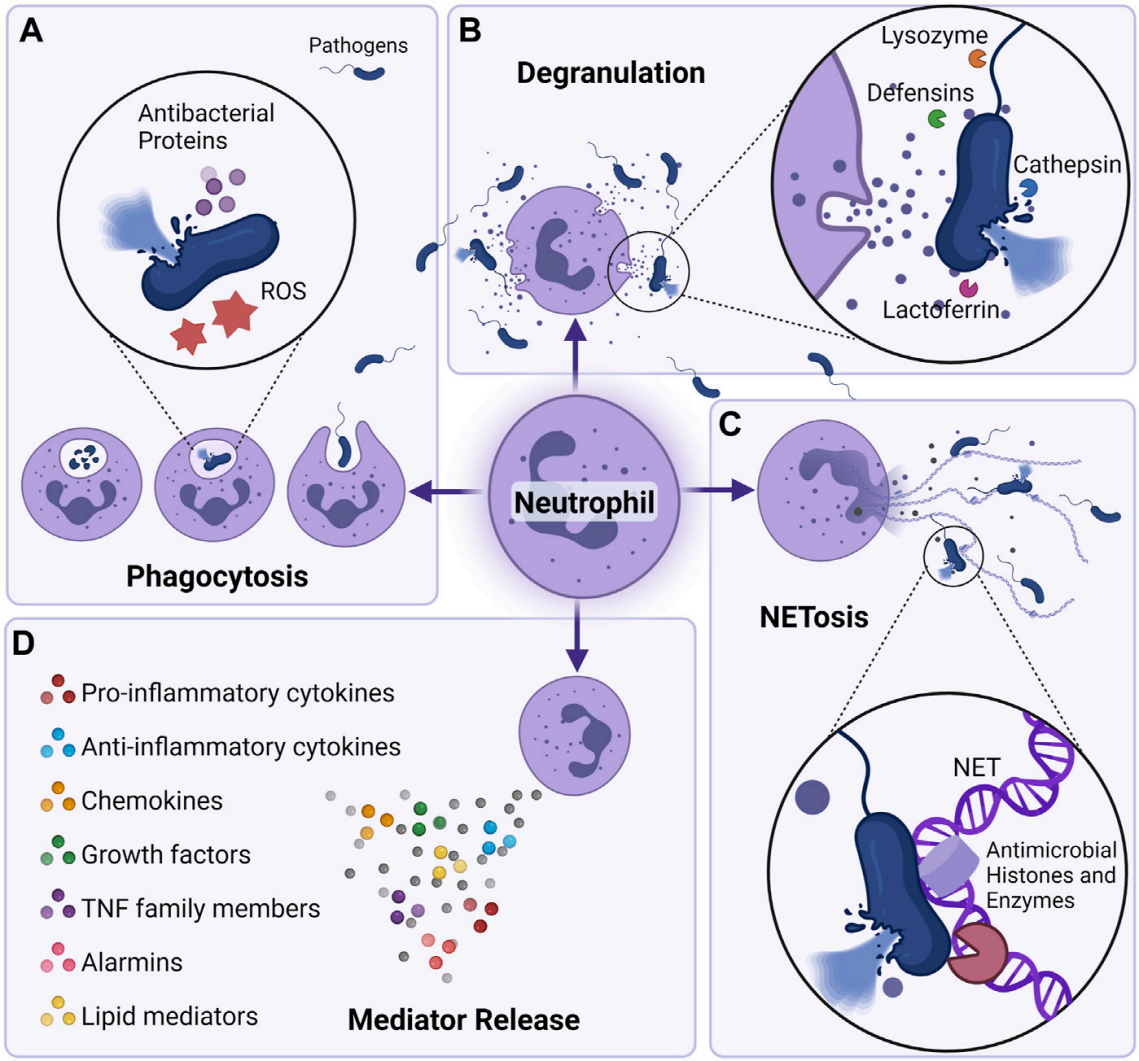
Neutrophil killing mechanisms. Neutrophils can eliminate pathogens intracellularly and extracellularly in a multitude of fashions. **(A)** Through phagocytosis, neutrophils are able to engulf and kill pathogens utilizing the actions of NADPH oxygenase-dependent mechanisms (reactive oxygen species) or antibacterial proteins (cathepsins, defensins, lactoferrin, and lysozyme) (Borregaard, 2010; Hager et al., 2010). **(B)** Neutrophils can also degranulate their antibacterial proteins to the extracellular milieu and cause killing of pathogens. **(C)** neutrophil can also eliminate pathogens by secreting neutrophil extracellular traps (NETs) (Brinkmann et al., 2004). Composed of core DNA elements to which histones, proteins and enzymes are attached, NETs immobilize pathogens and facilitate microorganism phagocytosis, they can also directly kill pathogens through the action of antimicrobial histones and proteases (Papayannopoulos and Zychlinsky, 2009). **(D)** Neutrophils can also shape their environment by secreting a plethora of soluble mediators that can skew the inflammatory response but also alter the function of immune cells themselves. Neutrophils are able to secrete pro (IL-1β, IL-6, IL-17, TNF-α) as well as anti-inflammatory cytokines (IL-1RA, IL-10, etc), chemokines (CXCL1, CXCL2, CXCL8, CXCL10, etc) growth factors (BAFF, G-CSF, M-CSF), alarmins (S100A8/A9, S100A12), and lipid mediators (resolvin, lipoxin) (Tecchio et al., 2014; Malengier-Devlies et al., 2021).

Neutrophils are the most abundant of all immune cells in the circulation. In humans, neutrophils comprise 50–70% of all circulating leukocytes, and in mice they account for 10–25% (Mestas and Hughes, 2004). Neutrophils are short-lived with a half-life of 9–18 h in mice (Hidalgo et al., 2019), and, although a lot of controversy has been allocated to human neutrophil half-life (Pillay et al., 2010), recent data suggests that neutrophil half-life in humans is 13–19 h (Lahoz-Beneytez et al., 2016). Because of their shorter lives, neutrophils are produced *en masse* in the bone marrow at a rate of approximately 10^7^ cells/day in mice and 10^11^ in humans under steady state conditions (Dancey et al., 1976; Summers et al., 2010). However, during severe infections and other inflammatory conditions where the demand for neutrophils is high, neutrophils can see their life span increased to up to 7 days (Colotta et al., 1992; Moulding et al., 1998; Hidalgo et al., 2019), and their production rates increased by more than 10-fold compared to their numbers in the steady state (Summers et al., 2010). Neutrophils belong to the innate branch of the immune system; they are powerful effector cells equipped with an array of tools that not only help them to recognize dangers, but also to respond effectively to the challenges posed by both external and internal stimuli (Liew and Kubes, 2019). Neutrophils are the first line of defense against any type of dysfunction, ranging from invading pathogens to sterile and chronic inflammation. They are equipped with a repertoire of receptors capable of identifying different types of pathogen-associated molecular patterns (PAMPS) and damage-associated molecular patterns (DAMPS), which affords them a wide range of coverage (van Rees et al., 2016). The *modus operandi* of neutrophils depends on the context and varies according to the nature and type of stimuli they encounter. Neutrophils actions range from simply engulfing and destroying the invading microbial pathogen (phagocytosis), to release of antimicrobial granules (degranulation), to production and release of reactive oxygen species (ROS) that damage and destroy the pathogens, to finally, neutralize pathogens through extrusion of decondensed chromatin and of the entire granular cargo to the extracellular space (Almyroudis et al., 2013). This unique form of cell-death is termed NETosis, or formation of neutrophil extracellular traps (NETs), and contributes to host defense against invaders (Burgener and Schroder, 2020) (Figure 1). While neutrophils are essential for the clearance of pathogenic and damage-associated threats, their unwanted presence and persistent activation in tissues may lead to tissue damage through the release of cytokines, proteases and other factors contained within their cytoplasmic granules (Caielli et al., 2012; Kolaczkowska and Kubes). In most cases of infection or sterile injury, neutrophils are the first immune cells to arrive on location and are responsible for the initiation of the inflammatory process. However, with the persistence of the inflammatory process, neutrophils become necessary as parts of the resolution of inflammation, the initiation of wound healing, and the return of the system to homeostasis (Kolaczkowska and Kubes; Puhl and Steffens; Ma, 2021). Neutrophils have also been shown to modulate the effects of the adaptive immune system by manipulating cytokines necessary for both B- and T-cell survival and activation (Karmakar and Vermeren., 2021; Kvedaraite, 2021). Therefore, identifying the precise cellular mechanisms that regulate neutrophil trafficking and activation is of the utmost importance, especially when it comes to manipulating the immune response for a successful therapeutic benefit.

To evoke an immune response, immune cells are recruited towards the site of inflammation. In order for these cells to reach any area within the body, a vast network of connections is necessary to deliver immune cells from their reservoirs to the appropriate inflamed location. Although blood vessels are essential for oxygen, nutrient delivery, and disposal of waste products, they are also an important web of highways for immune cell surveillance and trafficking. Neutrophils usually patrol the blood vessels for signs of danger. When tissue damage/infection occurs, the ensuing alteration in homeostasis generates a wide range of signals. Tissue resident hematopoietic and non-hematopoietic cells secrete cytokines and chemokines in response to the insult, thus establishing a chemoattractant gradient. As “leaders” of the innate immune response, neutrophils need to sense, prioritize, integrate, and then initiate a migratory pattern towards the source of damage/danger. In order for neutrophils to reach the affected sites, they exit the blood vessels by a process termed neutrophil extravasation (Filippi, 2019). This process involves a complex cascade of events with multiple interactions between leukocytes and endothelial cells. These steps consist of leukocyte capture, rolling, firm adhesion, diapedesis and abluminal crawling (Butcher., 1991; Springer, 1994). However, under homeostatic conditions and within special vascular beds, a population of neutrophils referred to as the “marginated” pool can be found in direct contact with the vascular wall. Marginated neutrophils make up half of all neutrophils present in the circulation and are in equilibrium with the other population of circulating neutrophils known as the demarginated pool. It is unclear how these different pools are initially established and how they attain a dynamic equilibrium. However, it is clear that under certain stress conditions the demarginated pool may become more prominent and contribute significantly to the pool of circulating neutrophils (McDonald et al., 2010; Ince et al., 2019). In this review, we aim to provide an updated view on the process involved in neutrophil recruitment and their trafficking behaviors, with a special focus on the mechanisms that regulate margination and demargination of neutrophils from the vasculature.

## Neutrophil Origins and Fates

**FIGURE 2:**
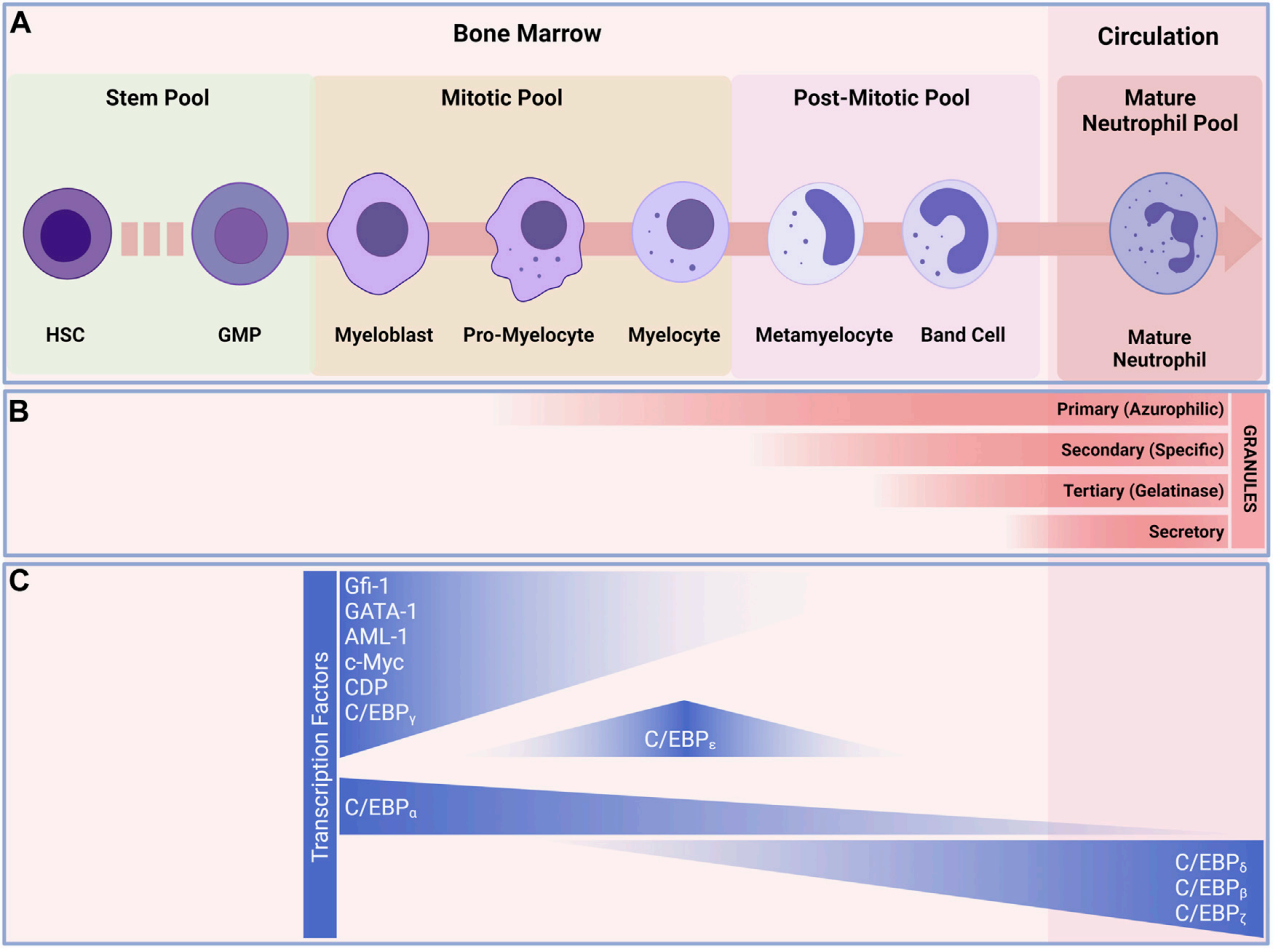
Granulopoiesis and development of neutrophils. **(A)** Under normal and steady state conditions, neutrophils are continuously generated in the bone marrow from myeloid precursors; they originate from hematopoietic stems and progenitor cells (HSPCs), go through a series of maturational steps spanning from mitotic pool, post-mitotic pool and leading to the formation of immature neutrophils in the bone marrow before maturing and released into the circulation. **(B)** During their development neutrophils develop granules that play key roles in neutrophil defense against invaders. Granulopoiesis begins with the appearance of Primary (Azurophilic) granules (e.g., Arginase-1, Cathepsin G, CD63, Defensins, Lysozyme, Myeloperoxidase, Neutrophil elastase) develop as neutrophils differentiate from myeloblasts to promyelocytes. Secondary (Specific) granules (e.g., CD15, CD66b, CR3(CD11/CD18), Lactorferrin, Lysozyme, neutrophil collagenase (MMP8), NGAL, OLFM4) form in myelocytes and metamyelocytes. Tertiary (Gelatinase) granules (e.g., ADAM-9, Gelatinase B (MMP9), Arginase-1, Ficolin-1) appear in band cells and secretory granules (i.e., CD10, CR1, chemoattractant receptors, CR3, C1q-R) are present in mature neutrophils. **(C)** transcription factors regulating neutrophils differentiation. The myeloblast is the first cell to adopt the neutrophil differentiation pathway, C/EBP-a favor granulocytic rather than monocytic differentiation. C/EBP-e constitute the other major neutrophil transcription factor and controls the pro-myelocyte to myelocyte transition. These transcription factors also play a major role in the formation of granules during the development of neutrophils. Abbreviations: HSC, Hematopoietic stem cell; MMP, multipotent progenitor; CMP, common myeloid progenitor; GMP, granulocyte/macrophage progenitor.

According to the current paradigm, neutrophil development starts with the granulocyte-monocyte progenitors (GMPs) and progresses through a continuum of maturation and differentiation stages (Yvan-Charvet and Ng, 2019) (Figure 2). The stages range from the mitotic pool of granulocyte-committed precursors, able to divide and composed by the first true neutrophil committed progenitor, the promyelocytes (Zhu et al., 2018). These cells can then either proliferate or differentiate into myelocytes (round nucleus and a less dense cytoplasm when compared to the promyelocytes) (Cowland and Borregaard, 1999; Zhu et al., 2018). Following the mitotic stage, the neutrophil progenitors lose their abilities to divide and enter the postmitotic pool. Progenitors in the postmitotic pool represent progenitors that are starting to undergo true maturation, beginning with the metamyelocytes (characterized by kidney-shaped nucleus and clear cytoplasm) (Dancey et al., 1976). Metamyelocytes then mature into a horseshoe-shaped nucleus and a clear cytoplasm (Dancey et al., 1976; Terashima et al., 1996). And finally the mature neutrophilic pool, where the neutrophil undergo the terminal differentiation in the bone marrow before being released into the circulation (Tak et al., 2013). The trafficking of neutrophils within the bone marrow (BM) is reliant on many retention and egress signals. The CXCR4/CXCL12 and VLA-4/VCAM-1 are the major pathways for neutrophils retention and egress from the BM. (Petty et al., 2009; Eash et al., 2010). In the BM neutrophils adhere to endothelial and stromal cells. During their maturation, neutrophil expression of CXCR4 decreases, leading to a lowered expression of VLA-4 (Rankin, 2010). Release of neutrophils from the BM requires the coordinated and combined actions of the CXCR4/CXCL12 pathway, together with signaling by CXCR2/CXCR2-Ligands (CXCL1, CXCL2, CXCL5 and CXCL6) (Sadik et al., 2011; Mendelson and Frenette, 2014). Osteoblasts are the major source of CXCL12, whereas CXCL1 and CXCL2 are constitutively expressed on endothelial cells in the BM (Mendelson and Frenette, 2014). The reciprocal interactions of CXCR2 with its ligands and CXCR4 with CXCL12 are what influence the trafficking of neutrophils from the BM to the systemic circulation. The neutrophils then leave the BM as freshly released neutrophils into the circulation, where they patrol and go through the process of aging, become aged neutrophils, and return to the BM for clearance.

The current views on neutrophils progenitors are under continuous evaluation, with recent advances in transcriptomics at the single-cell level and cell cycle-based and multiparametric flow analyses, the presence of early, intermediate, and late neutrophilic precursors with distinct gene and transcription factor signatures has been revealed (Velten et al., 2017; Evrard et al., 2018; Zhu et al., 2018; Dinh et al., 2020).

It is important to note that once maturation is complete, a portion of mature neutrophils are released into the circulation where they patrol the circulation for signs of perturbations, and infiltrate tissues where they reside, before returning to the bone marrow for recycling (Scheiermann et al.; Casanova-Acebes et al.). However, upon maturation, not all neutrophils are released from the bone marrow, and many are retained as reserves for upcoming potential immune events (Donohue et al., 1958; Craddock et al., 1960). Recent studies have shown that neutrophils retained in the bone marrow play a crucial role in supporting hematopoietic niches as well as hematopoietic stem cell functions (Chen et al., 2017; Kawano et al., 2017; Bowers et al., 2018; Wei and Frenette, 2018). Interestingly, terminally differentiated neutrophils that are released into the circulation, patrol for a couple of hours before they senesce and return to the bone marrow for clearance. In both mouse and humans, freshly released neutrophils are endowed with distinct phenotypic properties that gradually change over time, following the circadian oscillations (Adrover et al., 2019). Homeostatic removal of neutrophils from the circulation is mainly mediated by macrophages through efferocytosis in the liver, spleen and in the bone marrow (Summers et al., 2010; Gordy et al., 2011). In mice, a senescent neutrophilic phenotype is defined by an increase of their surface CXCR4 (Nagase et al., 2002; Martin et al., 2003; Furze and Rankin, 2008), a chemokine receptor that, and together with CXCL12 (secreted by bone marrow stroma cells), functions as a retention signal on neutrophils in the BM. Under homeostatic conditions, the processes of neutrophil release from the bone marrow (fresh neutrophils) towards the circulation and their subsequent exit out of the circulation (aged neutrophils) in the absence of inflammation are referred to as aging (Adrover et al., 2016). Aging phenotypic changes on neutrophils include the upregulation of CXCR4, which encourages aged neutrophils to return to the BM (Martin et al., 2003). Aged neutrophils also exhibit a downregulation of CD62L (L-selectin) (Casanova-Acebes et al., 2013), higher expression of CD11b, and CD49d and expression of other surface molecules such as TLR4, ICAM-1, CD11c (Zhang et al., 2015). The neutrophil aging process also comes with morphological changes (smaller cells containing fewer granules, and display of a granular multilobullar nucleus) (Casanova-Acebes et al., 2013). Following injury, infiltration of neutrophils to adequate tissues and orchestration of their functions leads to their exhaustion and eventual death. Clearance of these neutrophils *in situ* is a well characterized mechanism that is majorly conducted by resident macrophages (Savill et al., 1989), and to a small extent dendritic cells (Greenlee-Wacker, 2016). The whole equation shift gears during severe inflammatory responses. The need for neutrophils in this context is great; neutrophils are massively attracted to the site of injury, their life span is increased, their clearance decreased, and the high demands of newly generated neutrophils pushes the bone marrow into a state of “emergency granulopoiesis”, that sometimes extends to outside the BM in a process called “extramedullary myelopoiesis” (Manz and Boettcher, 2014; Wirths et al., 2014). Emergency granulopoiesis is well recognized in clinical settings by leukocytosis, neutrophilia and the increased release of immature neutrophils (myelocytes, metamyelocytes) into the circulation (Manz and Boettcher, 2014; Honda et al., 2016). During extramedullary myelopoiesis, neutrophils production may take place outside of the BM, primarily in the spleen, liver but also lymph nodes and lungs (Kim, 2010; Johns and Christopher, 2012; Lefrancais et al., 2017). Although out of the scope of this review, and has been provided in details elsewhere (Malengier-Devlies et al., 2021), it was noteworthy to mention the rapid adaptation of the hematopoietic system, switching to granulopoiesis and providing high-demand-neutrophils, during excessive inflammatory immune response scenarios.

Under steady state conditions, although their journey through the circulation is brief, neutrophils are present under two alternating states in the vasculature, either adhered to the vessel walls (marginated neutrophils) or freely circulating (demarginated neutrophils). Whether the marginated pool of neutrophils in the vasculature represents another reservoir for freely circulating neutrophils is still unknown. Despite the growing number of studies describing different and specialized functions and subsets of neutrophils, both in steady state and in inflammatory conditions, concrete evidence is lacking due to the absence of specific criteria used to define these subpopulations. More in-depth analyses such as deep phenotyping and next-generation sequencing (e.g., mass cytometry, single cell RNA) may be necessary to obtain a precise characterization, transcriptional profile, and function of highly purified mature and immature neutrophils in both homeostatic and inflammatory conditions.

## Neutrophil Heterogeneity

Neutrophils were once thought to be a homogeneous population. However, recent evidence suggests the existence of several different subsets of neutrophils (Ng et al., 2019). These subsets of neutrophils are shown to be specifically present in different conditions, such as chronic inflammation and cancer, in both humans and mice.

In the circulation, the heterogeneity of the neutrophil population is delineated by their fast-paced “ageing” process. This ageing of neutrophils is tightly regulated by a clock-driven mechanism called circadian oscillations (Scheiermann et al., 2012). Circadian rhythms affect key parameters in immune circulating cellular and humoral elements (circulating immune cells, hormones, and cytokines (Haus and Smolensky, 1999), as well in immune constituents in tissues (Scheiermann et al., 2012). These circadian rhythms oscillate depending on the resting and active phases of the species and are reliant on hormones (glucocorticoid and catecholamine) governed by the sympathetic nervous system (Dibner et al., 2010; Golombek and Rosenstein, 2010). This suggests that neutrophils adjust their functions in accordance with the changing demands of the day (Scheiermann et al., 2013). Two different phenotypes of neutrophils exist in the blood. The newly generated neutrophils, and the aged neutrophils, those two phenotypes of neutrophils have distinct properties and different functions (Adrover et al., 2016). Newly generated neutrophils are released from the bone marrow into the circulation during the resting phase. These fresh neutrophils are immuno-competent, they circulate the system, infiltrate tissues and are primed for inflammatory activities that arise during the active phase of the animal. During their journey, these fresh neutrophils undergo phenotypic changes in a process termed “ageing”; hallmarked by the loss of expression of certain surface markers (CD62L) (Van Eeden et al., 1997), and the gain in expression of others (CD11b, CXCR4). Aged neutrophils lose certain of their immuno-competent functions and are programmed to return to the bone marrow for recycling and termination by macrophages at the end of the active cycle (Martin et al., 2003; Casanova-Acebes et al., 2013; Adrover et al., 2019). Therefore, new generated neutrophils are released daily during the resting phase and are eliminated during the active phase, leaving room for fresh neutrophils release during the next cycle. Recent excellent reviews provide detailed overview of the circadian rhythms and their implications with both neutrophils as well as the immune system (Scheiermann et al., 2013; Aroca-Crevillen et al., 2020). When it comes to tissue-specific neutrophils, it has been shown that neutrophils indeed reside in several tissues under steady state conditions, hinting for the first time at true phenotypic diversity (Becher et al., 2014). This diversity in the neutrophilic population was uncovered through the analysis of different tissues and the expression levels of over 30 markers on neutrophils in tissues (Becher et al., 2014). Although the potential function of these so-called “residing” neutrophil populations has not been fully explored, there is evidence of neutrophils, for example, in the skin displaying “scout-like” functions. These patrolling neutrophils may allow for early detection of threats and recruitment of additional immune cells upon the first signs of danger (Ng et al., 2011). In the lungs, a large population of neutrophils is marginated in the microvasculature and has been shown to rapidly respond to infractions through CXCR4 signaling (Devi et al., 2013; Yipp et al.). Under steady state conditions, neutrophils may act as sentinel cells that patrol tissues. Under inflammatory conditions however, the neutrophil landscape enters a different level of diversification. The heterogeneity of neutrophils through conditions of inflammation, infection, chronic disease and cancer have been recently well documented (Silvestre-Roig et al., 2016; Seignez and Phillipson., 2017; Ley et al., 2018) and are beyond the scope of this review.

## Neutrophil Recruitment

Given the many functions neutrophils bring to the inflammatory process, the tightly regulated recruitment of these cells has been a major subject of research for many years. Recruitment of neutrophils to the site of inflammation follows several specific patterns. Neutrophils exit the bone marrow and wander freely within the circulation, interact with the vessel wall, roll along the activated endothelium and finally transmigrate to the inflamed tissue (Nourshargh and Alon, 2014). However, the infiltrating neutrophil population is viewed as a double-edged sword. Their key functions are essential for removal of any foreign threat but are also needed to initiate the resolution of inflammation and tissue repair mechanism. On the other hand, neutrophils can also be the cause of severe organ failure by damaging tissues through secretion of cytokines, proteases, other factors contained in their cytoplasmic granules and through their potential to activate the adaptive immune system (e.g., T and B cells).

As the neutrophil’s role as a modulator of the immune response becomes increasingly more evident, it is vital to fully understand neutrophil migration patterns in both chronic and acute inflammation. There are two distinct migration patterns for neutrophils-forward migration, which occurs during initial recruitment of neutrophils, and reverse migration, which relates to the migration of neutrophils away from inflamed sites (Nourshargh et al., 2016).

## Forward Migration

Forward migration is a process by which neutrophils migrate towards the source of inflammation. Neutrophils express a wide variety of surface receptors capable of sensing a vast array of signals that guides them to the site of injury. In most cases of inflammation, the initial signal is danger associated molecular patterns (DAMPs) (Pittman and Kubes, 2013) released from damaged and necrotic cells. DAMPs are sensed by either toll like receptors (TLRs) or/and NOD like receptors (NLRs) on the surface of neutrophils. DAMPs include DNA, proteins, N-formyl peptides, extracellular matrix components, ATP and uric acid. For example, N-formyl peptides such as fMet-Leu-Phe (fMLP) derived from bacterial proteins or mitochondrial products after tissue damage can activate neutrophils by binding to the fMLP receptor (FPR) 1, 2, or 3 (Raoof et al., 2010; Zhang et al., 2010; Li et al., 2016). In mice, FPR1 blockade results in suppression of neutrophil recruitment to the site of injury (McDonald et al.). Other signals produced by tissue and resident cells in response to injury, such as chemokines and lipid mediators, can also recruit neutrophils to the site of inflammation. Chemokines, such as the CXCL8 family, signaling through their G-Protein Coupled Receptors (GPCRs), CXCR1 and CXCR2 receptors, can activate a downstream signaling pathway that regulates neutrophil migration (Sai et al., 2008; Neel et al., 2009; Russo et al., 2014).

Lipid mediators can play a part in both neutrophil recruitment, as well the amplification of this recruitment. Lipid mediators are usually the products of metabolized arachidonic acid. The most prominent lipid mediator involved in neutrophil recruitment is leukotriene B4 (LTB4). LTB4 signals through the GPCR, LTB4-receptor on the surface of neutrophils. LTB4 can be secreted by neutrophils to stabilize their polarization or used to recruit new neutrophils to the site of inflammation (Afonso et al., 2012). Further amplification of the neutrophil recruitment pathways is usually complex and relies on a feedback loop. For instance, in a model of inflammatory arthritis, neutrophils recruited through LTB4 are activated and produce chemokines, such as CXCL2 and IL-1β, which in turn activate endothelial cells and macrophages to produce more chemokines and enhance the neutrophil recruiting process (Chou et al., 2010). Activated neutrophils also secrete metalloproteinases (MMPs) that cleave collagen present in the extracellular matrix and release collagen derived peptides that can serve as a neutrophil chemoattractant (Afonso et al., 2013). In other types of inflammation, such as that found in infection, in addition to tissue damage generated by the infection itself, the release of DAMPs, and PAMPs create another source of danger signals that recruit neutrophils. Although, in case of an infection, it is most likely the tissue resident immune cells, such as macrophages (Schiwon et al., 2014), dendritic cells (Sacramento et al., 2015), and mast cells (Abraham and St John, 2010), that recognize danger and pathogen signals and secrete adequate chemokines for the recruitment of neutrophils. The different subsets of neutrophils in both the circulation and tissues may also serve as an impartial factor in determining the recruitment of additional immune cells, including neutrophils.

## Reverse Migration

It is important for the host to have successful inflammatory response with minimal collateral damage. This is achieved through a rapid resolution process and early return to a state of homeostasis. The return to homeostasis is usually enabled by local resolution of inflammation through the removal of neutrophils from the site of injury (Soehnlein and Lindbom, 2010).

Clearance of neutrophil can be achieved in one of several possible ways, apoptosis, necrosis (Buckley et al.), and efferocytosis. However, the clearance of neutrophils does not necessarily come in the shape of apoptosis at the site of inflammation. Studies have shown that radiolabeled neutrophils that entered inflamed tissue were able to leave and return to the main circulation without undergoing apoptosis at the site of injury (Hughes et al., 1997). This process was termed neutrophil reverse migration (Mathias et al.). Reverse migration was first described in zebrafish larvae, in which it was shown that not all neutrophils die at the site of injury, but most recruited neutrophils eventually leave and traffic to distal sites post injury (Mathias et al., 2006; Yoo and Huttenlocher, 2011). Buckley and others (Buckley et al., 2006), described the ability of human neutrophils to reverse transmigrate through an endothelial monolayer *in vitro*; these neutrophils were phenotypically different and showed high expression of ICAM1 and low expressions of CXCR1, a phenotype that was found in the peripheral blood of patients with systemic inflammation (Buckley et al., 2006). In addition, neutrophil that have presumably undergone reverse migration *in vitro* are less susceptible to apoptosis and produce more ROS (Buckley et al., 2006). As neutrophils involved in reverse migration are usually recruited from tissues where they first encounter injury, much debate exists on the nature of their inflammatory signature and phenotypes. Neutrophils expressing reverse migration markers (ICAMhi and CXCR1low) during the physiopathology of sepsis were found to present a pro-inflammatory phenotype, are highly active, have prolonged lifespan (Zhang et al., 2015; Ng et al., 2019), delayed apoptosis (Ng et al., 2019), and exhibit high production of superoxide (Ode et al., 2018), as well as high levels of inducible nitric oxide synthase (iNOS) and NETs (Hesse et al., 2004). The question remains whether reverse-migrated neutrophils can adopt an anti-inflammatory repertoire. Future studies will surely bring better understanding and uncovering of the different phenotypes reverse-migrated neutrophils can adopt during their journey.

Several studies have been established since then, revealing more details about reverse migration in zebrafish (Robertson et al., 2014; Ellett et al., 2015), mice (Duffy et al., 2012; Owen-Woods et al., 2020), and even in human neutrophils (Hamza et al., 2014). An important question related to reverse migration is the outcome of those neutrophil in the system. This is an important aspect as systemic inflammation after severe trauma can lead to multiple organ failure in patients (Tsukamoto et al., 2010). In zebrafish, neutrophils that have reverse migrated can be found in the circulation several days after leaving the wound (Yoo and Huttenlocher, 2011). Higher numbers of neutrophils carrying the reverse migration phenotype are found in the circulation in humans with acute pancreatitis that also develop acute lung injury (Wu et al., 2016). In mice, the genetic deletion of junctional adhesion molecule C (JAMC) allows for a decreased expression of JAMC after injury at the endothelial junction, and thus increases the occurrence of neutrophil reverse migration (Colom et al., 2015). In this mouse model, induction of an acute pancreatitis increased the level of reverse migrated neutrophils in the circulation, which lead to increased severity of lung injury and systemic inflammation (Wu et al., 2016). Recently, our lab has shown that during acute myocardial infarction, the NLRP3 inflammasome-primed neutrophils upregulate CXCR4 and reverse migrate to the BM, where they release IL-1β that enhances granulopoiesis and fuels the injury with more neutrophils (Sreejit et al., 2021).

Reverse migrated neutrophils have also been shown to travel and localize to the lymph nodes (Hampton et al., 2015), or the bone marrow (Duffy et al., 2012), modulate lymphocyte proliferation, and alter the immune responses. Taken together, these studies suggested neutrophil reverse migration as a new approach to resolve inflammation, and possibly a new therapeutic, especially in diseases hallmarked by heavy neutrophil infiltration. However, one of many caveats of this approach is that activated reverse migrated neutrophils are redistributed to other locations and organs within the body, contributing to further spreading of the inflammation. Furthermore, many aspects of reverse migration require much work to be done, for instance, the fate of reverse migrated neutrophils, the occurrence of this mechanism in human disease, and the exact mechanism through which reverse migration happens remain unclear.

Just as PMNs recruitment to the sites of inflammation require several steps, such as capture, rolling, firm adhesion and *trans*-endothelial migration, reverse migration is a continuous and multistep process. Nourshargh et al. (Nourshargh et al., 2016), proposed various terminologies to describe the different processes through which reverse migration can occur, including reverse abluminal crawling (rAC), reverse interstitial migration (rIM), reverse luminal crawling (rLC), and reverse *trans*-endothelial cell migration (rTEM).

The precise mechanism that underpins reverse migration of neutrophils is yet to be identified, but several mechanisms have been proposed. Competition between chemoattractant sources, neutrophil downregulation/desensitization of specific chemokine receptors, decreased levels of chemoattractant, endothelial permeability, and neutrophil intrinsic transcriptional changes are amongst the mechanisms that are proposed to explain neutrophil reverse migration. Recently, Wang et al. (Wang et al., 2017), used photoactivatable-GFP (PA-GFP) neutrophils to track their final destination during inflammation. They reported and revealed that 24 h after injury, neutrophils had left the site of injury and were found in the lungs and bone marrow. The reverse migrating neutrophils found in the bone marrow showed increased expression of CXCR4 (Buckley et al., 2013; Nourshargh et al., 2016; Wang et al., 2017), indicating a markedly strong migration pattern towards the bone marrow. Another proposed mechanism that may affect neutrophil reverse migration is the ability of inflammation to affect endothelial permeability. For instance, inflammation damages the endothelial junctions, thus increasing endothelial permeability and leakage. The leakage of chemokines from the site of inflammation creates a secondary chemotactic gradient that confuses neutrophils to reenter the circulation (Marki and Ley, 2020; Owen-Woods et al.). Other studies have speculated that loss of sensitivity to chemokine cues forces neutrophils to move in a reverse direction. For example, it has been shown that during reverse migration neutrophils have downregulated expression of the chemokine receptor CXCR1 (Buckley et al., 2006), and are thus unable to respond to the main chemokines responsible for neutrophil migration. Chemotactic repellent is another mechanism hypothesized to be implicated in neutrophil reverse migration (de Oliveira et al., 2016). CXCL8 functions as a chemoattractant at lower concentrations, at higher concentrations, though, it acts as chemorepellent (Tharp et al., 2006). In addition to these, other mechanisms have been proposed for reverse migration (Elks et al., 2011; Nourshargh et al., 2016; Ji and Fan, 2021). Although an interesting and promising pathway, many questions remain unanswered in the process of reverse migration and more research must be conducted before we fully uncover the mechanistic details behind this orchestrated movement of neutrophils in the context of inflammation.

## Marginated and Demarginated Pool of Neutrophils

The concept that circulating granulocytes do not constitute the entirety of the intravascular leukocyte population dates as early as 1867, when Cohnheim (Cohnheim, 1867) observed white blood cells in a marginal position along the walls of the vasculature. (Mauer et al., 1960), in 1960 first conducted an experiment to identify the number of granulocytes in the circulation. The study showed that 50% of 32P-labelled granulocytes injected into healthy subjects could not be traced after infusion. Later on, it was shown that the number of neutrophils can be increased by treating subjects with adrenaline (Athens et al., 1961). The authors concluded that the total blood granulocyte pool was twice as large as the pool calculated from the blood volume, and that this pool consisted of two compartments: the circulating granulocyte pool and the marginal granulocyte pool. The size of the individual marginated pool can be calculated by measuring the mean intravascular transit time through the organ and its blood flow (i.e., the prolonged transit of neutrophil through an organ’s capillary bed) (Peters et al., 1985; Ussov et al., 1995).

Several investigations showed the existence of variable neutrophil transit times in different organs such as the lungs (Hogg and Doerschuk, 1995), spleen (Peters et al., 1985), liver and bone marrow (Ussov et al.). It is unclear the reasons behind different transit times for neutrophils in organs, a process that might be linked to marginated neutrophils being educated differently through various types of organs, and thus serving divergent functions (Rosales, 2018). Furthermore, it is unknown why marginated, mature neutrophils persist in these organs. One possible explanation is that they serve as a reservoir for rapid deployment in response to organ specific infection or injury. Another plausible explanation explores the possibility of these neutrophils in fulfilling some important immuno-organ related functions. For instance, in the spleen, which represents an important secondary lymphoid organ and an important site for the development of adaptive B-cell immune responses, Puga and others described two populations of splenic neutrophils located in the vicinity of marginal zone B-cells (Puga et al., 2011). These populations of splenic neutrophil B cell-helper 1 and 2 (NBH1 and NBH2) induce T-cell independent B-cell activation and elicit class switching, somatic hypermutation and production of immunoglobulins through the release of B-cell specific factors such as BAFF, april and IL-12. In addition to these populations, two additional neutrophil populations were recently identified in the spleen. These populations showed different expression levels of the Ly6G protein that were identified during *Streptococcus* pneumonia infection (Deniset et al., 2017). One of the major reservoirs of marginated neutrophils is the lung. Neutrophils in the lungs are usually found in the alveolar capillaries (Doerschuk et al., 1987), and it is thought that the longer transit time of neutrophils through the lungs is because of the deformation neutrophils undergo due to their larger diameter when compared to the smallest capillaries. The delayed transit time might also account for their role in patrolling the lungs for any sign of infection. In extreme conditions, the marginated pool of neutrophil in the lungs also serves to replenish the circulating population (Yipp et al.). Another explanation for neutrophil margination proposed is that adherent or marginated cells are in the process of exiting the circulation to migrate towards injury sites (Ley et al., 2007).

**FIGURE 3:**
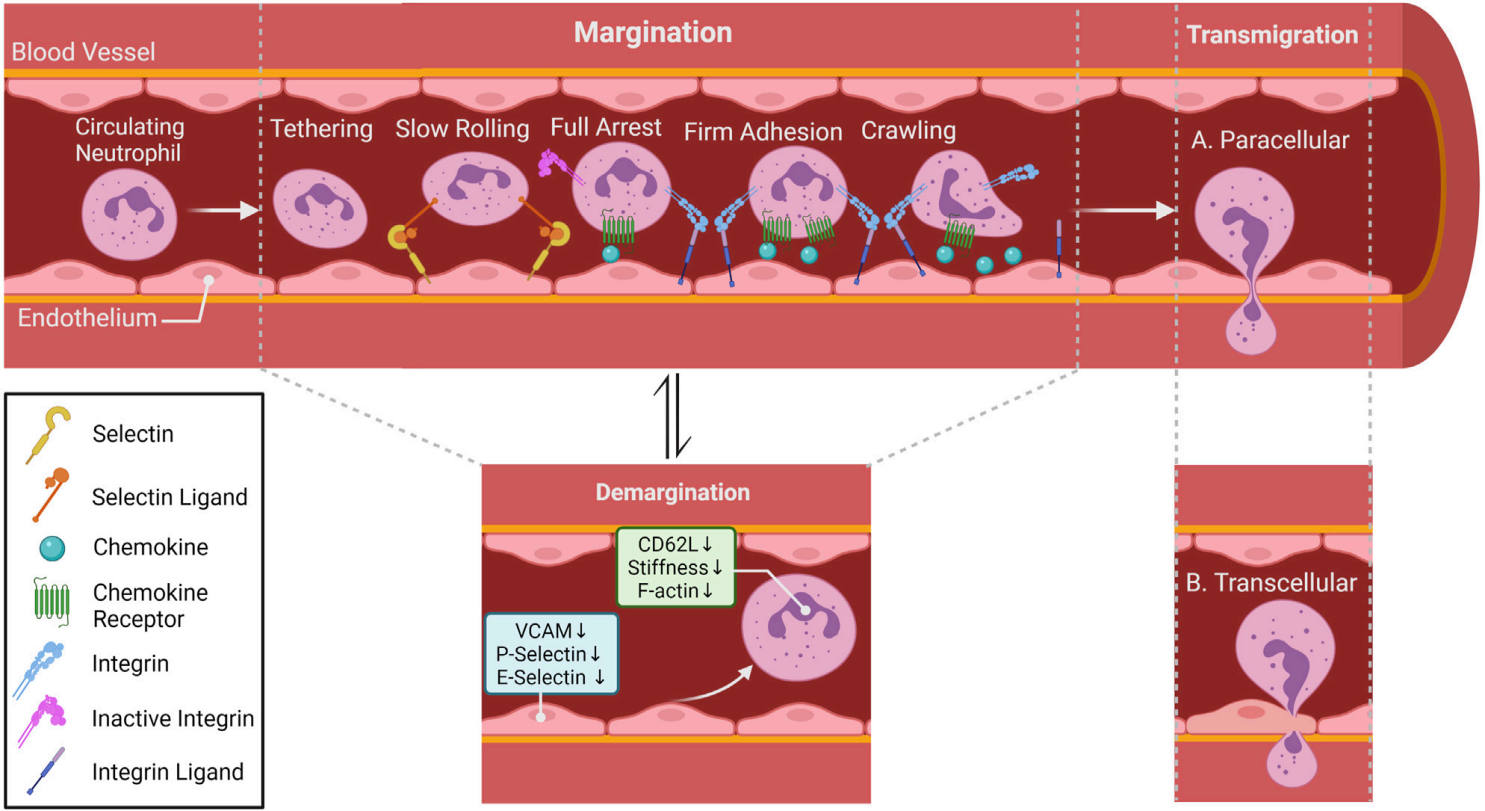
Neutrophil migratory patterns. In most tissues, neutrophil recruitment cascade involves tethering, rolling, full arrest, adhesion crawling and finally migrate into tissue by paracellular (between endothelial cells; **(A)** or transcellular transmigration (through endothelial cells)); **(B)** Rolling is mostly selectin-dependent, whereas adhesion, crawling and transmigration depend on integrin interaction. Chemokines lining the luminal part of the endothelium activate neutrophil surface integrin expression allowing neutrophils to follow the chemokine gradient along the endothelium. Within special vascular beds (lungs and the liver), neutrophils under steady state conditions can be found in direct contact with endothelial cells, this population of neutrophil is referred to as the marginated or intravascular retained pool. Catecholamines and glucocorticoids can alter surface adhesion molecules (Trabold et al., 2010a; Margaryan et al., 2017), as well as induce conformational changes in cytoskeleton (Fay et al., 2016) of neutrophils allowing them to detach from the vasculature and become the demarginated pool of neutrophils.

Under homeostatic conditions, it is well established that the circulating neutrophils are in equilibrium with the marginated neutrophils. However, whether margination is an active or a passive process is still under debate. What is interesting to note is the assumption that the marginated pool of neutrophils consists of non-activated neutrophils interacting with non-activated endothelial cells (ECs). Thus, the concept of classical adhesion cascade that occurs during inflammatory processes might not apply in the context of margination. Indeed, several studies have shown that certain adhesion molecules are not necessary for neutrophil margination (Doerschuk, 2001; Kolaczkowska and Kubes, 2013). However, intra-vital microscopy (IVM) studies conducted on mouse lungs showed that neutrophils under steady state conditions possess migratory behavior similar to the adhesion cascades observed in neutrophils under inflammatory conditions (i. e., tethering, crawling and firm arrest) (Yipp et al., 2017). Intravital microscopy also revealed that neutrophil patrol unstimulated draining lymph nodes of different organs including the lungs (Lok et al., 2019; Bogoslowski et al., 2020). Taken together, it is safe to deduce the uncertainty of what constitutes or drives the marginated pool of neutrophils. What is even more confounding is the existence of the circulating pool of neutrophils in the circulation. The demargination process is another key event that is not well characterized or explored. What is known about circulating neutrophils (demarginated neutrophils) is that this process is prevalent following strenuous exercise, smoking, stress, and ingestion of food. Even though the mechanism of demargination is not completely understood, certain factors such as stress hormones, in particular glucocorticoids and catecholamines can alter the different stages of the adhesion cascade (Figure 3). Catecholamines, such as epinephrine and norepinephrine, are an important class of systemic immune modulators that are released systemically by the adrenal gland and locally by sympathetic nerves. Depending on the duration of their signaling, catecholamines have either immune enhancing or immune suppressive effects (Dhabhar, 2008). The effects of norepinephrine and epinephrine are modulated by GPCRs called adrenergic receptors and are classified into three major types, α1, α2, or β adrenergic receptors. β receptors are further divided into β1, β2, and β3 receptors all of which are expressed on neutrophils (Dhabhar, 2009).

Stress hormones can affect leukocyte and, more specifically, neutrophil migratory properties *via* diverse mechanisms (Méndez-Ferrer et al., 2008; Dhabhar et al., 2012). A recent publication provided evidence that catecholamines can induce rearrangement of the cellular cortical actin in human granulocytes, decrease their rigidity, reduce their stiffness, and lead to their demargination (Fay et al., 2016). This could explain the rapid increase in granulocyte abundance triggered by these hormones in the absence of mobilization from reservoir tissues. Additionally, catecholamines can alter cytokine levels and expression of adhesion molecules on the surface of immune and endothelial cells (Trabold et al., 2010b; Scanzano et al., 2015a).

In addition to their direct effects on leukocyte adhesion, catecholamines can also modulate functions of tissue resident immune cells, such as macrophages. As major producers of cytokines, these immune cells are largely responsible for the effect of catecholamines on cytokine levels (Flierl et al., 2009). Epinephrine and norepinephrine have been shown to directly activate NF-κB in isolated peritoneal macrophages, causing them to release pro-inflammatory cytokines such as TNFα, CXCL12, IL-1β, and IL-6. In a murine skin wound model, tissue resident macrophages produce IL-6 in response to chronic β2 adrenergic receptor activation (Kim et al., 2014). IL-6 decreases L-selectin levels on the surface of neutrophils and enhances their demargination capabilities (Suwa et al., 2000). The continuous activation of macrophages and IL-6 production leads to sustained neutrophil trafficking to the site of injury (Ulich et al., 1989; Kim et al., 2014); a potential mechanism that might explain long-term stress and its association with delayed wound healing.

Although β2 adrenergic receptors have been shown to be the most commonly expressed adrenergic receptors on leukocytes, mRNA for other adrenoceptor subtypes are also present on immune cells (Scanzano and Cosentino, 2015). α2 receptors are known to play important roles in the expression of surface adhesion molecules (Scanzano et al., 2015a). Experiments using pharmacologic agonists for the α2-receptor reduced the trafficking of human neutrophils by inhibiting CD62L shedding and inhibition of CD11b increased expression (Herrera-García et al., 2014). The same experimental approach also showed a detrimental role for endothelial cells by upregulating an array of adhesion molecules favoring neutrophil migration, indicating that both neutrophils and the endothelium are important targets for catecholaminergic signaling in the regulation of neutrophil trafficking. In addition to stress hormones, multiple inflammatory mediators such as leukotriene B4, complement component C5a, IL-8 and tumor necrosis factor-α (TNF-α) have also been shown to promote neutrophil demargination; although in this case the increase in circulating neutrophils was attributed to release from the bone marrow sinusoids (Terashima et al., 1998).

Glucocorticoids are another class of stress hormones that can influence leukocyte migration. The systemic elevation of glucocorticoids is known to attenuate the inflammatory and cellular immune responses to tissue injury and infection (Hübner et al., 1996; Padgett et al., 1998). Much like catecholamines, glucocorticoid administration favored leukocyte demargination (Fay et al., 2016), and patients given dexamethasone showed significant evidence of leukocytosis in their circulation. *In vitro*, dexamethasone treatments have also been shown to induce demargination, independent of changes in vascular adhesion molecule expression, *via* inducing changes to the actin cytoskeleton of granulocytes and enabling their detachment (Fay et al., 2016). *In vivo*, dexamethasone injection causes alterations to endothelial cell expression of adhesion molecules. In addition to the biophysical properties, glucocorticoid also modulate the expression of key receptors on neutrophil surface to influence their maturation, homing, and egress. For example, neutrophil maturation is accelerated in rats treated with mifepristone (a glucocorticoid receptor antagonist) (Cavalcanti et al., 2007), an effect attributed to the reduced expression of Annexin A1 in neutrophils. Annexin A1 is upregulated by glucocorticoids, and the loss of Annexin A1 in mice leads to higher levels of CXCR4 expressing neutrophils labeled as having an “aged” phenotype (Machado et al., 2016). In addition, Annexin A1 deficient mice were reported to have deficiency in homing to CXCL12. Taken together, the inability to efficiently return to the bone marrow and increased maturity leads to neutrophilia and might be another explanation to the mechanisms through which glucocorticoid is involved in neutrophil demargination. In the context of inflammation, glucocorticoids are administered to inhibit the immune response through glucocorticoid receptor activation that limits the expression of many pro-inflammatory cytokines. In a model of LPS induced inflammation, dexamethasone treatment resulted in reduced circulating leukocyte counts, as well as rolling, adhesion and emigration, whereas the use of mifepristone increased adhesion and emigration (Gregory et al., 2009). The literature is limited when it comes to the exact mechanisms that alter neutrophil adhesion and promote their demargination. Many risk factors that increase neutrophil numbers in the circulation are being explored. For instance, smoke-exposed neutrophils are significantly less adherent and fail to increase their adherence following stimulation with fMLP. Examination of neutrophil surface markers in these experiments showed no alteration in expression of adhesion molecules (Selby et al., 1992). Another *in vitro* study indicated that short-term exposure of neutrophils to smoke caused shedding of surface L-Selectin (CD62L) (Ryder et al., 1998). This change in L-selectin levels could be the cause of neutrophil loss of adhesion and subsequent demargination.

Stress hormones exert diverse effects on granulocytes migration under steady state and inflammatory conditions. These effects are dependent on not only the affected cell type but also, the location, duration and source of the stress hormone signal, as well as the inflammatory scenario and environment. Epinephrine, norepinephrine, as well as glucocorticoids have been shown to induce neutrophilia in circulation. Although the mechanisms through which stress induce demargination are still not fully understood, therapies using synthetic agonists and antagonists directed at alleviating the extent of recruitment of neutrophils are already utilized in the clinic.

## Neutrophils in Cardiovascular Diseases

The implication of the immune system in the development of CVD has been widely explored and appreciated (Epelman et al., 2015; Swirski and Nahrendorf, 2018). With advances in the field of neutrophil biology, neutrophils have moved from being considered to be bystanders and biomarkers of CVD to actual modulators and mediators of the cardiovascular inflammation and repair scenery.

Many risk factors aid in fueling of CVDs. Metabolic factors such as lipid and glucose metabolism, lifestyle factors such exercise stress, and nutrition, all have been known to play a critical part in CVDs. Regardless, the more pressing question is whether these risk factors modulate the immune system, and more specifically neutrophils, into perpetuating cardiovascular physio-pathologies.

One of the major attributes of CVD risk factors is that they are able to alter the inflammatory responses by reprogramming hematopoietic stem and progenitor cells (HSPCs), directly or indirectly. The functional disturbance of HSPCs leads to increased and sustained neutrophil numbers and promotes chronic inflammation. In humans, neutrophils counts are a strong predictor of future cardiovascular events (Friedman et al., 1974). After myocardial infarction (MI), the number of neutrophils in the circulation is directly correlated to the infarct size, decline in left ventricular ejection fraction, and heart failure development (Arruda-Olson et al., 2009; Chia et al., 2009; Dogan et al., 2009). In mice, the number of circulating neutrophils positively correlates with the size of their atherosclerotic lesions (Drechsler et al., 2010). Altered lipid (hypercholesterolemia) and glucose (hyperglycemia) metabolism can have major effects on myelopoiesis. In mice, increased accumulation of cholesterol in the cell membrane, as a consequence of defective cholesterol efflux, induces HSPCs proliferation and a differentiation towards the myeloid cell lineage (Yvan-Charvet et al., 2010). Hypercholesterolemia also fuels the process of neutrophil production in mice by regulating production of IL-23 by macrophages that leads to systemic release of granulocyte colony-stimulating factor (G-CSF) (Westerterp et al., 2012; Casanova-Acebes et al., 2018). Much like hypercholesterolemia, hyperglycemia was shown to manipulate HSPCs quiescence and proliferation (Nagareddy et al., 2013; Takubo et al., 2013). Hyperglycemia activates neutrophils and promotes their release of S100A8 and S100A9 alarmins, which interact with myeloid progenitor cells, and Kuppfer cells, driving myelopoiesis (Nagareddy et al., 2013), and IL-6 mediated thrombocytosis (Kraakman et al., 2017) respectively, and help accelerate atherogenesis and atherothrombosis. As we age, HSPCs accumulate somatic mutations that leads to clonal expansion of hematopoietic stem cells without hematologic malignancy. This process is known as clonal hematopoiesis of indeterminate potential (CHIP), and has been associated with increased risk of CVDs (Jaiswal et al., 2017; Dorsheimer et al., 2019). CHIP is linked to loss of function mutations in certain genes (*DNMT3A, TET2, and ASXL1*) and gain of function of other genes (*JAK2* ^*V617F*^) in myeloid lineages. In an atherosclerosis-prone mouse model, partial bone marrow reconstitution with TET2 HSPCs was sufficient for their clonal expansion and led to marked increase in atherosclerotic plaque size (Fuster et al., 2017). In another study, BM expressing JAK2-V617F was transferred into hypercholesterolemic mice, and this increased neutrophil infiltration in early atherosclerotic lesions, increase spontaneous NET release and led to accelerated atherogenesis in these mice (Wang et al., 2018). Stress is another cardiovascular risk factor (Kivimaki and Steptoe, 2018). individuals who have experienced stressful events have a higher risk of myocardial infarction, stroke, arrhythmias and arterial thrombosis (Remch et al., 2018; Song et al., 2019), with prevalent increases in the number of circulating neutrophils (Heidt et al., 2014). Stress can influence the release of stress hormones (glucocorticoid and catecholamine), that increase proliferation of hematopoietic progenitors. When atheroprone mice were subjected to chronic stress, sympathetic nerve fibers released surplus of noradrenaline, which triggered HSPCs proliferation, and decreased bone marrow niche secretion of CXCL12 levels through β3-adrenergic receptor. This led to increase numbers of neutrophil and monocytes in the circulation and in atherosclerotic lesion, and thus exacerbated inflammation and promotion of atherosclerosis (Heidt et al., 2014).

In atherosclerosis, neutrophils are actively implicated in the recruitment of monocytes (Alard et al., 2015), the promotion of adhesion molecules on the surface of endothelial cells, the regulation of endothelial cell permeability (Rasmuson et al., 2019), modulation of the fate and function of macrophages (Soehnlein et al., 2008; Delporte et al., 2014), and the shaping the overall atherogenic immune environment, and plaques instability. Pharmacological inhibition of NETs release by neutrophils through protein-arginine deaminase type 4 (PAD4) (an enzyme essential in NET formation), resulted in reduced atherosclerosis development (Knight et al., 2014; Liu et al., 2018). Furthermore, NETs contain DNA-cathelicidin-related antimicrobial peptide (CRAMP) complexes. CRAMPs trigger production of interferon-α by plasmacytoid dendritic cells that further aid in amplifying atherosclerosis (Doring et al., 2012). Neutrophils NETs can also trigger production of proatherogenic cytokine IL-1β by macrophages (Warnatsch et al., 2015). This may be of clinical relevance as canakinumab (anti IL-1β) has been shown to reduce CVD.

Neutrophils have also been implicated in the instability of atherosclerotic lesions. Analysis of both human and mice arterial intima shows higher neutrophil numbers that correlated with signs of plaque instability (Silvestre-Roig et al., 2019). These plaques are typically lipid-rich, dominated by macrophages, with large necrotic cores and fibrous caps composed of vascular smooth muscle cells (VSMCs) and collagen (Libby et al., 2019). Activated VSMCs stimulate NETs formation through the effect of CCL7. Rich with histone H4, these NETs promote to the formation of pores in the plasma membrane of VSMCs leading to their death. The death of VSMCs leads to thinning and instability of the fibrous cap (Silvestre-Roig et al., 2019).

During Myocardial infarction (MI), attracted by cell debris and inflammatory mediators released by activated resident cells, neutrophils infiltrate the ischemic myocardium in large quantities (Puhl and Steffens, 2019). Shortly after ischemia occurs, resident immune cells and cardiomyocytes begin to produce inflammatory cytokines and chemokines such as IL-1, IL-6, TNF and CCL-2 (Gwechenberger et al., 1999; Frangogiannis, 2004); cardiac fibroblasts release hematopoietic growth factors such as GM-CSF (Anzai et al., 2017). In conjunction with CCL2 and CCL7, these events trigger a massive production and recruitment of neutrophils and monocytes to the circulation and to the ischemic hearts. Neutrophils initially recruited to the infarcted regions aim to phagocyte and clear dead cell debris caused by the ischemia. However, they concomitantly cause collateral cardiac damage by the release of reactive oxygen species (ROS), NET formation, as well as secretion of extracellular vehicles (EVs), and inflammatory mediators (Carbone et al., 2013). However, beyond their role as pro-inflammatory and damage inflicting cells, it has been recently shown that neutrophils are also necessary for the anti-inflammatory, pro-angiogenic, and the reparative effects, and thus being beneficial in cardiac wound healing (Peiseler and Kubes, 2019; Puhl and Steffens, 2019). Neutrophil depletion, following MI in mice, impaired cardiac functions, increased fibrosis and led to heart failure (Horckmans et al., 2017). This dual role of neutrophils in damage and healing raises the question of possible neutrophil subsets that might provide these different and opposite functions. The heterogeneity of neutrophils was reported in the context of cancer (Fridlender et al., 2009) and inflammation (Tsuda et al., 2004). Two major categories of neutrophil subsets emerged, the N1 subset that exhibits pro-inflammatory markers (*Ccl3, Il-1β, Il-12a, and Tnf-α*), and the N2 subset that was anti-inflammatory and pro-tumorigenic (*Cd206* and *Il-10*). Using gene expression profiling following MI, Ma et al. (Ma et al., 2016), were able to identify both N1 and N2 neutrophils in the infarct region, with N1 phenotype appearing immediately after injury and the reparative N2 phenotype appearing 5–7 days post-MI. The heterogeneity of neutrophils during MI was further divided into several subtypes. Using a proteomic approach, neutrophil proteome was observed to shift into 4 different subsets depending on their function. Day 1 neutrophils exhibiting high degranulation with increased MMP activity, day 3 neutrophils showed upregulation of apoptosis and induction of extracellular matrix (ECM) organization, day 5 neutrophil further increased their ECM reorganization profiles, and day 7 neutrophils had reparative signature (Daseke et al., 2019).

While many aspects of neutrophil involvement in CVDs are well characterized and extensively reported (Swirski and Nahrendorf, 2018; Silvestre-Roig et al., 2020; Gopalkrishna et al., 2021), many questions remain unanswered. For instance, during MI, what is the source of the first responding neutrophils? Several evidences suggest that the first waves of neutrophils arriving to the ischemic hearts are from the BM reservoirs at least 12 h after induction of MI (Sreejit et al., 2020). However, given the fact that neutrophils are found in ischemic hearts prior to 12h, that those neutrophils are responsible for causing the majority of damage to the myocardium, and the fact that the reservoir in the BM are mostly consisting of premature neutrophils, another source of neutrophil maybe more probable. The mature marginated pool of neutrophils that resides in the circulation can be easily and rapidly recruited to the source of injury and might be the initial supplier of immune cells to infiltrate the hearts and initiate inflammation. Whether marginated, or demarginated pools of neutrophils have actual implication in CVDs is not known. Since stress causes demargination and has been shown to be a risk factor for CVDs. It is highly possible for stress to cause demargination and recruitment of marginated neutrophils to the ischemic heart.

## Neutrophils, a Drug Target

Being the first immune cells that infiltrate any site of injury or infection, neutrophils have the potential to be a key modulator of the inflammatory response. Failure to appropriately resolve inflammation can have disastrous effects. The excessive and persistent infiltration of neutrophils into tissues has a role in several inflammatory diseases, including myocardial infarction (Silvestre-Roig et al., 2020), rheumatoid arthritis (Wright et al., 2014), pulmonary fibrosis (Cantin et al., 2015), and multiple organ failure (Tsukamoto et al., 2010). Neutrophils and their biological functions have, therefore, been highlighted as potential targets for drug therapies. However, complete neutrophil depleting therapies resulted in mortality and/or severe immunodeficiency in both humans and mice. Therefore, neutrophil migratory patterns, especially those targeting the initial and destructive phases of neutrophil recruitment, are attractive targets for anti-inflammatory therapies. Targeting primary signals in the recruitment of neutrophils, colchicine, a microtubule inhibitor, inhibits neutrophil recruitment to site of inflammation *in vivo*. Colchicine is used to treat human conditions including gout, chronic pericarditis and familial Mediterranean fever (Cocco et al., 2010), and has recently emerged as an agent to reduce cardiovascular risk in the eye-catching LoDoCo2 trial. (Cocco et al., 2010). Some pathways targeting neutrophils focus on fine-tuning the neutrophilic response without eliminating it; this is achieved by targeting chemokine receptor antagonists (Lazaar et al., 2011; Moss et al., 2013). Other compounds target lipid mediator-induced inflammation with LTB4R antagonists (Dalli et al., 2013). Alternative approaches to the conventional methods targets and promotes neutrophil reverse migration away from the site of injury/inflammation. However, targeting this pathway raises a couple of questions regarding the fate of the reverse-migrated neutrophils (Schwab et al., 2007; Robertson et al., 2014). With the recent advance in the field of neuro-immunology and the prominent role in which stress affects the immune system, one therapy of perhaps more promising results comes from targeting stress hormones induced migration of neutrophils. Not surprisingly, following myocardial infarction (MI), the number of neutrophils in the circulation directly correlate to both the infarct size and a decline in left ventricular ejection fraction (Chia et al., 2009). Furthermore, a major contributor to cardiomyocyte apoptosis following MI injury are the numbers of infiltrating neutrophils to the ischemic hearts (Vinten-Johansen, 2004; He et al., 2018). As discussed throughout this review, neutrophils are not only vital in clearance of pathogens and debris, but also in the resolution of the inflammatory processes (Kolaczkowska and Kubes, 2013). The engulfing of apoptotic neutrophils by macrophages in the inflamed tissues activates an anti-inflammatory response characterized by the production of IL-10, TGFβ as well as pro-resolving lipid mediators (Frangogiannis, 2012). In a chronic mouse MI model induced by permanent left anterior descending (LAD) coronary artery ligation, long-term neutrophil depletion led to severe worsening of cardiac functions, increased fibrosis, and progressive increase in biomarkers associated with heart failure (Horckmans et al., 2017). These findings demonstrate the long-term effects of neutrophil in initiating resolution of inflammation. Consequently, timely therapy strategies aimed at reducing the initial scourge of neutrophils to the infarcted hearts, while maintaining their long-term effect on resolution are indispensable. From this perspective, the use of β-adrenoceptor antagonists (β-blockers) in cardiovascular disease (CVD) have led to increase in both survival rates and life expectancy (Lloyd-Jones et al., 2010). In addition to reducing heart rate, and blood pressure as well as anti-arrhythmogenic and anti-ischemic effects (Gheorghiade et al., 2003), β-blockers exert their functions on the immune system and, more specifically, on the infiltrating and migratory patterns of neutrophils to the ischemic hearts (Garcia-Prieto et al., 2017; Clemente-Moragon et al., 2020).

Although colchicine and metoprolol are cheap and commonly used drugs in daily practice, they both have a distinct pattern of side effect, and have several clinical contra-indications. Furthermore, although their benefit in several conditions is obvious, their efficacy is limited and unlikely to be fully optimize neutrophil derived contributions to pathology.

Harnessing the immune system is therefore a great way to introduce new and improved therapies throughout the plethora of diseases. Targeting one of the first cells to infiltrate injury might bring new insight to the whole spectrum of immune modulatory aspects that ensues. Although the full biology of neutrophils is not fully explored, daily discoveries in the field are bringing us closer every day to more personalized and targeted, as well as individualistic, therapies.

## Conclusion

The definition of the neutrophil as an innate immune cell once characterized by simple and singular function has dramatically changed in the last few decades. Being the first immune cell type at sites of injury or infection, it has the potential to be a key moderator of the immune system and play defining role in controlling the pathological outcomes of inflammation.

Remarkable progress has been made in understanding the mechanisms that regulate neutrophil activation, their role in inflammation and their migratory patterns. Nonetheless, many caveats still exist in understanding the full spectrum of biological functions of these immune cells. More importantly, aspects of migration, such as demargination, are not fully understood. It is still not known if margination or demargination are “good” or “bad” during the inflammatory process. In case of inflammation, we know that margination is necessary as part of the neutrophil adhesion cascade for migration towards the source of injury. However, why margination occurs in the absence of any stimuli (steady state) is still unanswered. If the phenomenon of margination is important for the initiation of the adhesion cascade, what is the purpose of demarginated neutrophils? It is known that stress can alter the adhesion state of neutrophils. A plausible explanation is that demargination is an evolutionarily conserved mechanism that may represent an overreaction to an anticipated injury or damage to the host. More research is needed to uncover the full mechanism of margination and demargination of neutrophils in disease, as well as in steady state.
